# Construction and experimental verification of the spatial attitude kinematic model of advanced support equipment

**DOI:** 10.1038/s41598-022-22869-8

**Published:** 2022-10-26

**Authors:** Hanzhao Chen, Kun Zhang, Zhengxian Sun, Chengjun Hu, Yuxia Li, Xuntao Wei, Mingchao Du, Ya Liu, Xin Wang

**Affiliations:** 1 Shandong Provincial Key Laboratory of Robotics and Intelligent Technology, Shandong University of Science and Technology, Qianwangang Road 579, Qingdao, 266590 Shandong China; 2 University of York, Heslington, York YO10 5DD UK; 3 Shandong Energy Group, Jingshi Road 10777, Jinan, 250014 Shandong China; 4 Beidou Tiandi Co., Ltd, Xibu Road 2, Xian, 710000 Shaanxi China; 5 China Coal ( Tianjin ) Underground Engineering Intelligent Research Institute Co., Ltd, Dafeng Road, Tianjin, 300120 China

**Keywords:** Engineering, Physics

## Abstract

The roadway in coal mine is prone to deformation of the surrounding rock under the influence of complex disturbance, which induces the imbalance of support attitude matching between roadway surrounding rock and advanced coupling support equipment, breaks the mechanical balance of the original coupling support system, and causes safety accidents. The postures and offsets of the various working spaces of the advanced hydraulic supports under the influence of complex disturbances are systematically analyzed. The D–H parameter matrix transformation method is used to establish the spatial posture kinematic model of a single/group of advanced hydraulic supports. The posture parameters of each key node are obtained through global coordinate transformation. Based on the comparison of the working attitude parameters of the support obtained through simulation analysis and model calculation, the average absolute error of the support height calculation results of the single group of advanced hydraulic support attitude model is 3.92 mm, and that of the coordinate calculation results of the advanced hydraulic support group is (3.26 mm, 1.99 mm), which meet the accuracy requirements of the advanced hydraulic support attitude monitoring. Finally, the spatial attitude kinematic model of a single/group of advanced hydraulic supports is verified and analyzed by using the advanced hydraulic support attitude monitoring test bench. The maximum error value of the experimental results is 4.9 mm, which verifies the accuracy of the established spatial attitude kinematic model of the advanced hydraulic support. The research results can provide theoretical and technical support for the intelligentization of the advanced coupling support system and the safe and efficient mining of coal mine.

## Introduction

The advanced hydraulic support group, as the main support equipment of the fully mechanized working face, together with the roadway surrounding rock-anchor support system, constitutes the advanced coupling support system, which plays a role in the safe and stable support of the mining roadway^1,2^. The stress of the surrounding rock of the mining roadway increases with the continuous increase in mining depth, and the complex disturbance easily induces deformation of the surrounding rock, such as floor heave, roof subsidence, and two-sided deformation. This phenomenon causes the advanced hydraulic support in the working process of coupling support to be in the abnormal supporting position, destroys the stability of the advanced coupling support system, and induces equipment damage and roof caving and other safety accidents^3,4^. Therefore, how to establish the space attitude model in the working state of the advanced hydraulic support and realize the dynamic monitoring of the attitude has become an urgent problem to be solved.

In view of the support characteristics and posture monitoring technology of the advanced hydraulic support group coupling support system, many experts and scholars worldwide have carried out a significant amount of research and achieved remarkable results. Chen et al.^5^ established the kinematic measurement model of an advanced hydraulic support. They also proposed the calculation method of yaw and roll angles of the advanced hydraulic support based on ultrasonic ranging data. Vaze et al.^6^ measured the length and angle of the column and connecting rod in the support process with an encoder and an inclination sensor and calculated the attitude parameters that are difficult to determine in the hydraulic support by using the established mathematical model. Ren et al.^7^ studied the pressure sensor, attitude sensor and other advanced sensors and integrated them into the existing hydraulic support electro-hydraulic control system, forming a comprehensive perception system of hydraulic support. Ferreras et al.^8^ took the hydraulic support as the research object and studied the support process and the state of the hydraulic support in the process of moving. The relationship between the acceleration of the corresponding components was obtained by changing the motion speed of the executive components. Bai et al.^9^ studied the coupling relationship between the support and the surrounding rock, established the attitude mathematical model of the support, analyzed the correlation between the motion parameters of the actuator of the support and the support height, and proposed the control method of the support and the selection of the sensors. Zhang et al.^10^ studied the characteristics of tilt sensor attitude measurement, proposed a fusion method of multi-sensor data, created a support attitude model to solve attitude angle and support height, developed a set of hydraulic support attitude monitoring platform, and realized remote monitoring of the hydraulic support. Xu et al.^11^ studied the attitude measurement principle of the attitude measurement unit and inclination sensor and designed a set of position and attitude monitoring system for hydraulic support. They used a variety of data processing algorithms to improve the measurement accuracy of the sensor and solved the key attitude parameters of hydraulic support by combining the mathematical model and the neural network.

In the aspect of attitude monitoring sensor development and fusion technology. Liu et al.^12^ studied the characteristics of a three-axis gyroscope, acceleromete, and magnetometer and developed a high precision MEMS attitude sensor. Xu et al.^13^ studied the measurement characteristics of attitude sensors and found that accelerometers and magnetometers can continuously correct the measurement results of gyroscopes. Kalman filter was used to fuse the data of three sensors, and the static and dynamic characteristics of attitude measurement unit were improved. Luo et al.^14^ studied the measurement of yaw angle of the measured object with a gyroscope and an accelerometer and realized the monitoring of vehicle attitude and speed during steering.

The above-mentioned research results provide theoretical and engineering practice support for the adaptive support control, attitude monitoring method, and advanced safe and efficient support theory of the advanced coupling support system. However, the existing support attitude monitoring technology is mostly focused on the research direction of the working face hydraulic support, and research on the spatial attitude monitoring of the advanced hydraulic support in the limited space is scarce. In particular, the monitoring of the spatial attitude change of the advanced hydraulic support under the influence of the surrounding rock deformation has not been studied. Furthermore, the existing advanced hydraulic support attitude monitoring technology focuses on the sensor selection and fusion, and the construction of the spatial pose kinematic model of the working attitude of the advanced hydraulic support after the influence of complex disturbances is not perfect. On this basis, this work systematically analyzes the change form of spatial attitude in the working process of the advanced hydraulic support under the influence of complex disturbance.

The above-mentioned research results provide theoretical and engineering practice support for the adaptive support control, attitude monitoring method, and advanced safe and efficient support theory of the advanced coupling support system. However, the existing support attitude monitoring technology is mostly focused on the research direction of the working face hydraulic support, and research on the spatial attitude monitoring of the advanced hydraulic support in the limited space is scarce. In particular, the monitoring of the spatial attitude change of the advanced hydraulic support under the influence of the surrounding rock deformation has not been studied. Furthermore, the existing advanced hydraulic support attitude monitoring technology focuses on the sensor selection and fusion, and the construction of the spatial pose kinematic model of the working attitude of the advanced hydraulic support after the influence of complex disturbances is not perfect. On this basis, this work systematically analyzes the change form of spatial attitude in the working process of the advanced hydraulic support under the influence of complex disturbance. The D–H parameter matrix transformation method is used to establish the spatial attitude kinematic model of a single/group advanced hydraulic support. The global coordinate transformation is used to obtain the attitude parameters of the key nodes of the advanced hydraulic support, and the accuracy of the established spatial attitude model is verified by comparing the model solution results with the simulation results. Finally, the corresponding experimental test is carried out on the space attitude monitoring test bench of the advanced hydraulic support to verify the correctness of the space attitude model. The established spatial attitude model of advanced hydraulic support will provide theoretical basis and technical support for the dynamic monitoring of spatial attitude of advanced support equipment in the limited space under the influence of surrounding rock deformation and the safe and efficient mining of coal mines.

## Working pose and offset analysis of the advanced hydraulic support group

The stress of adjacent roadway along the roadway increases due to the poor working environment and the increase of mining depth, causing deformation of the surrounding rock, such as floor heave and subsidence, in the roadway floor. Consequently, the advanced hydraulic support will be in the abnormal support position in the working process. Moreover, the support in the abnormal support position repeatedly support the roof, which will cause the deformation of the roof and surrounding rock of the roadway and roadway collapse, resulting in safety accidents. Therefore, this work comprehensively examines the deformation of the various surrounding rocks caused by the complex disturbance of the roadway surrounding rock and analyzes the possible postures of the advanced hydraulic support after disturbance and impact in the working process.

### Pose offset analysis of the single group advanced hydraulic support

1. Base in the horizontal support posture
  Under ideal conditions, the base should be near the roadway floor, and the top beam should also be as close as possible to the roadway roof during the support process of the advanced hydraulic support. If the bottom plate has not deformed, then the base is still in a horizontal posture. In the actual working conditions, a coupling relationship exists between the top beam and the surrounding rock. The roof deformation is induced by complex disturbances, such as mining and rock movement, which will make the top beam present three postures of “horizontal”, “head up”, and “low head”, as shown in Fig. 1.

**Figure 1:**
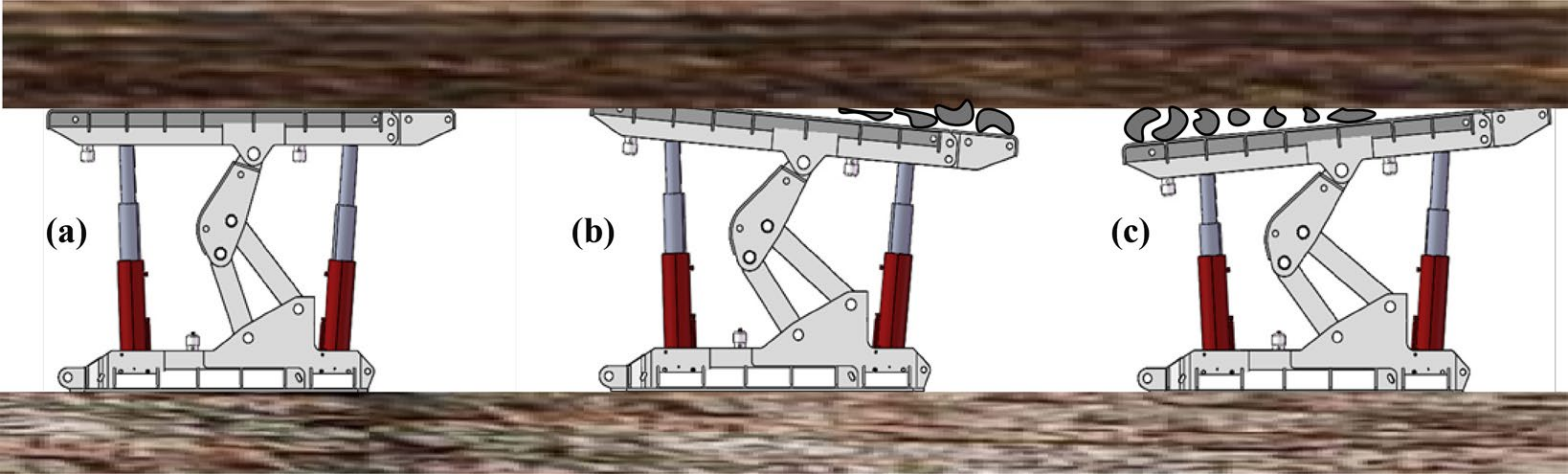
Three postures of the advance hydraulic support when the roadway floor is horizontal: (**a**) ideal supporting posture; (**b**) base “horizontal” and top beam “head up”; and (**c**) base “horizontal” and top beam “low head”.
2. Base in the subsidence support posture
  Floor subsidence will occur as a result of the irregular movement of floor strata during the support process of the advanced hydraulic support. At this time, the support demonstrates a “low head” posture along its propulsion direction, and the top beam presents “horizontal”, “head up”, and “low head” three postures, as shown in Fig. 2.

**Figure 2:**
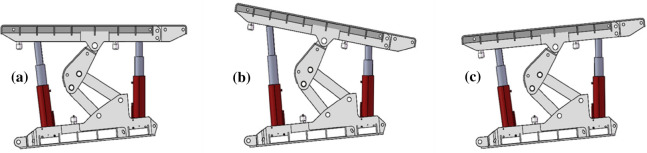
Three postures of the advance hydraulic support when the roadway floor sinks: (**a**) base “low head” and top beam “horizontal”; (**b**) base “low head” and top beam “head up”; and (**c**) base “low head” and top beam “low head”.
3. Base in the drum support posture
  During the support process of the advanced hydraulic support, the rock movement of the floor cause floor heave of the roadway. The advanced hydraulic support presents the posture of “head up” along its advancing direction. At this time, the top beam still presents three postures of “horizontal”, “head up”, and “low head”, as shown in Fig. 3.

**Figure 3:**
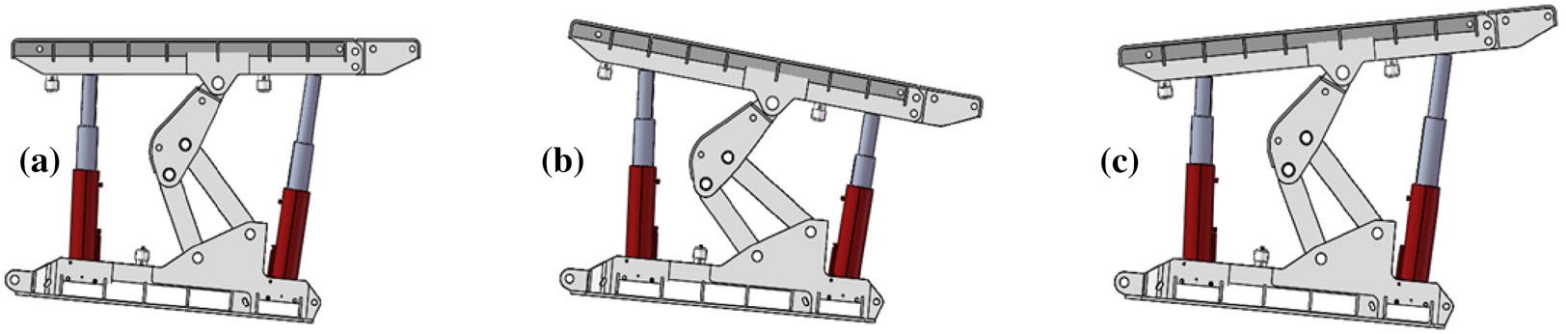
Three postures of the advance hydraulic support when the roadway floor is bulging: (**a**) base “head up” and top beam “horizontal”; (**b**) base “head up” and top beam “head up”; and (**c**) base “head up” and top beam “low head”.

During the support process of the advanced hydraulic support, the rock movement of the floor causes floor heave of the roadway. The advanced hydraulic support presents the posture of “head up” along its advancing direction. At this time, the top beam still presents three postures of “horizontal”, “head up”, and “low head”, as shown in Fig. 3.

### Position offset analysis of the advanced hydraulic support group

The advanced hydraulic support group is composed of the left and right two separate supports. A group of supports will have a relative deviation due to the deformation of the surrounding rock during the support process, thus affecting the overall stability of the support group. The deviation mode between the support groups is shown as follows.

1. Spacing deviation of the left and right bracket groups
  During the support process of the advanced hydraulic support, the advanced hydraulic support needs to balance the contraction of the cylinder due to the deformation of the roadway on both sides of the roadway to maintain a safe distance with the two sides of the roadway. At this time, the distance between the left and the right advanced hydraulic support groups will gradually reduce, and the moving angle needs to be timely adjusted. If the angles are not timely adjusted, then it will lead to collision extrusion of the two supports, resulting in damage to the hydraulic support, as shown in Fig. 4.

**Figure 4:**
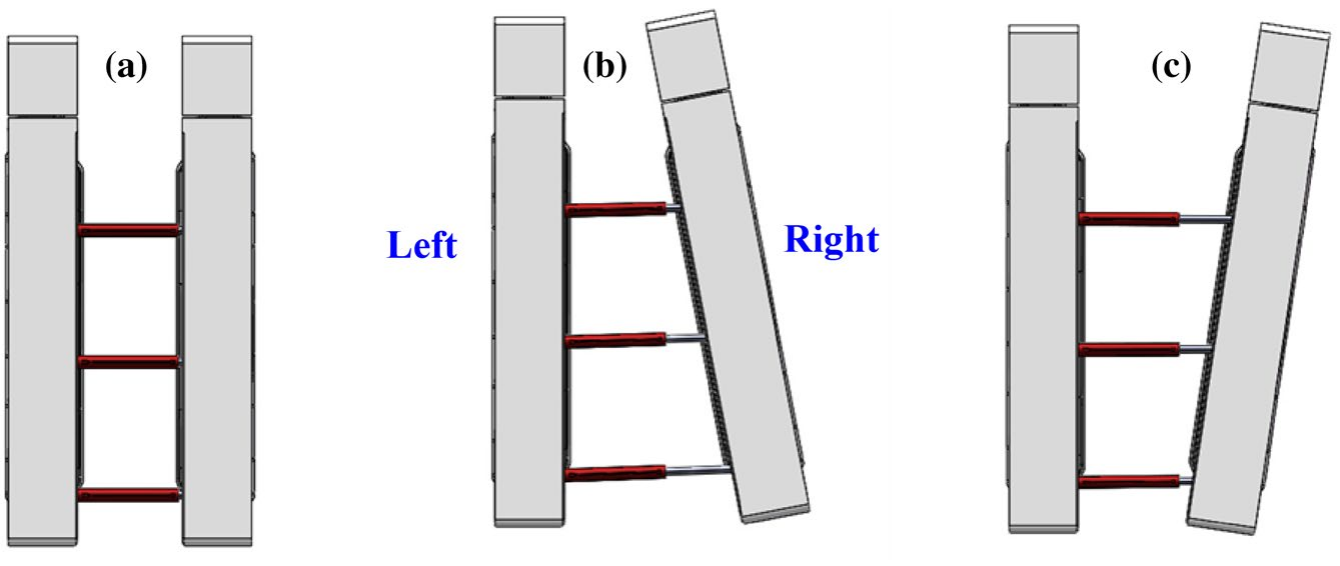
Left and right spacing deviation of the advance hydraulic support: (**a**) Ideal supporting state; (**b**) non-ideal supporting state (left avertence); and (**c**) non-ideal supporting state (right avertence).
2. Anterior and posterior spacing offsets of the stent group
  During the support process of the advanced hydraulic support, the two supports are in the same plane, and an offset between the front and the rear spacing may be detected due to the complex and changeable situation of the roadway floor and the influence of the stroke of the pushing cylinder used in the pushing phase. The amount of the pushing cylinder must be timely adjusted to ensure that the advanced hydraulic support group is in a straight line; otherwise, it will cause error accumulation and affect the support effect, as shown in Fig. 5.

**Figure 5:**
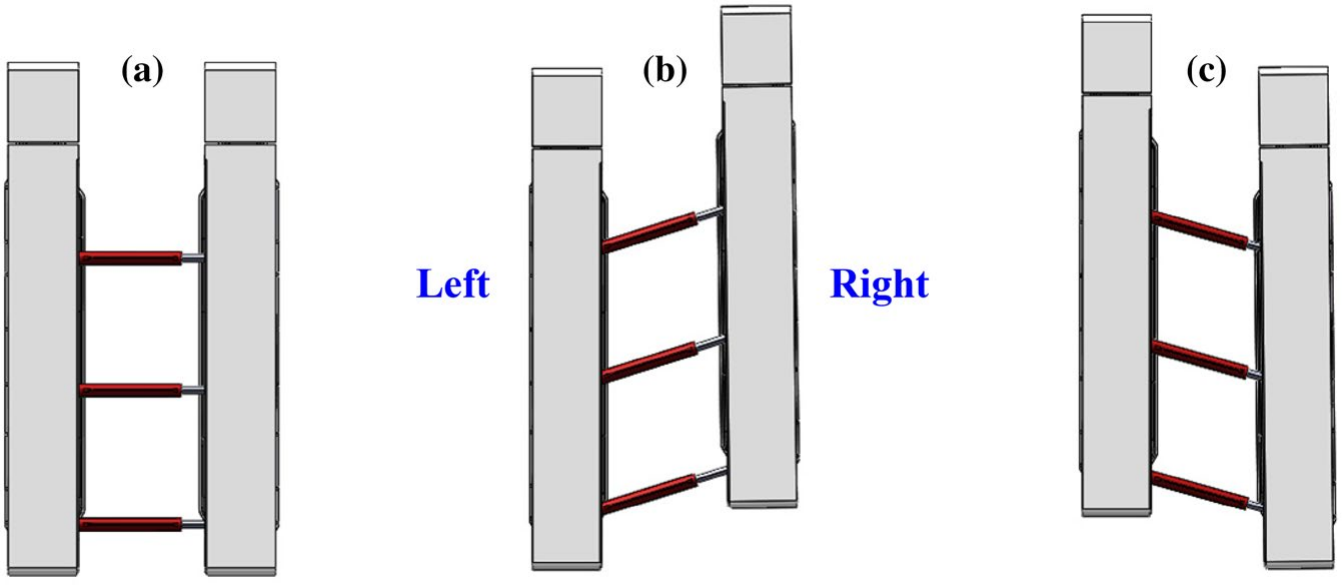
Offset of the front and rear spacing between the leading hydraulic supports: (**a**) ideal supporting state; (**b**) non-ideal supporting state (forward lead); and (**c**) non-ideal supporting state (retrusion).
3. Upper and lower spacing offsets of the support group
  During the working process of the advanced hydraulic support, the support is inclined to left or right due to the local deformation of the roadway floor or roof, which will affect the support stability of the advanced hydraulic support group. If the deviation angle is considerably large, then it will lead to the dumping of the advanced hydraulic support, as shown in Fig. 6.

**Figure 6:**
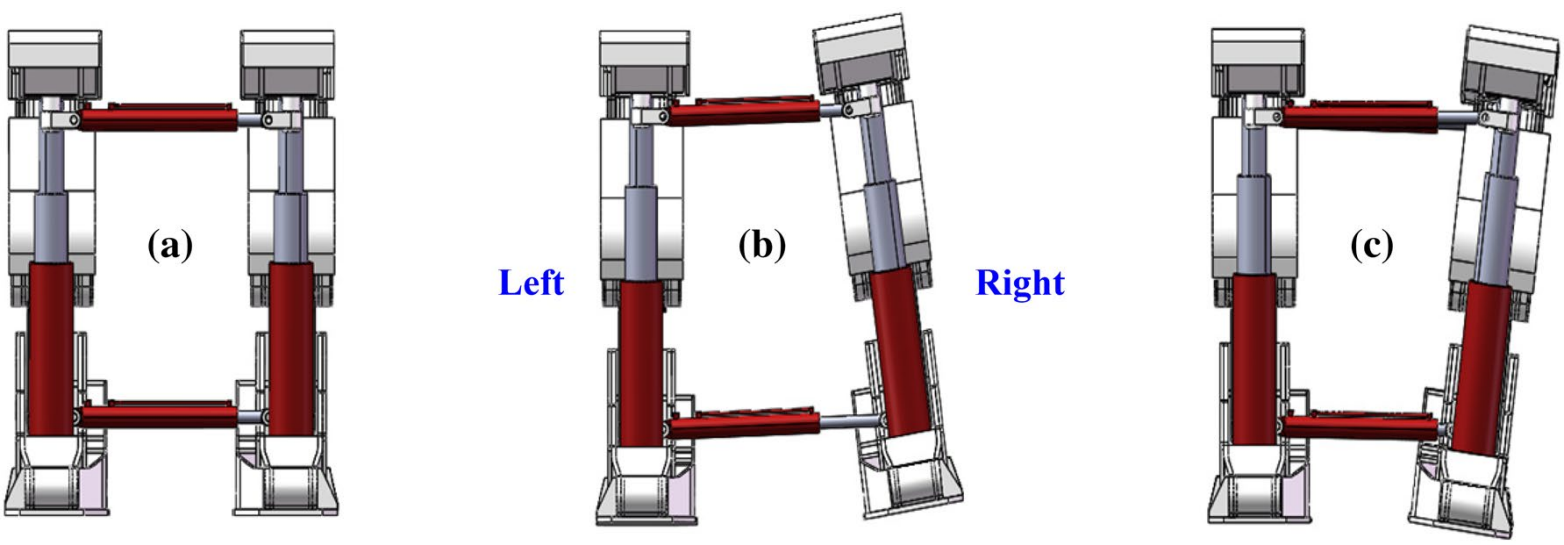
Upper and lower spacing deviation of advance hydraulic support: (**a**) ideal supporting state; (**b**) non-ideal supporting state (left bank); and (**c**) non-ideal supporting state (right bank).

## Spatial pose kinematics model construction of the advanced hydraulic support group

Accurate pose monitoring in the working process of the advanced hydraulic support is one of the key theoretical and technical problems that restrict the realization of intelligent and unmanned mining in a fully mechanized working face. The kinematic model of the single group of the advanced hydraulic support and that of the advanced hydraulic support group are established to better study the posture of each component, analyze the motion relationship between the components, and consider the various postures caused by the deformation of the surrounding rock in the working process of the advanced hydraulic support. The posture parameters of the key nodes of each component are solved by coordinate transformation and geometric analysis, which provide data basis for the design of the posture monitoring system of the advanced hydraulic support.

### Kinematic model of the single group of the advanced hydraulic support

**Figure 7:**
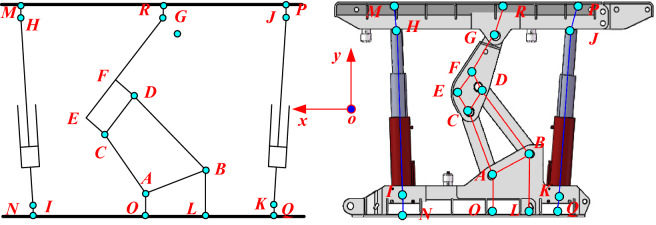
2D model of the advance hydraulic support.

The motion model of a single group of advanced hydraulic supports is simplified to carry out the kinematic analysis of a single group of the advanced hydraulic support. The simplified 2D model diagram can intuitively show the connection form of each actuator, as shown in Fig. 7. The kinematic equation of the single group of the advanced hydraulic support is established, and the pose parameters of key nodes are solved using the global coordinate system of the cross-section in combination with D–H matrix and geometric analysis method.

**Figure 8:**
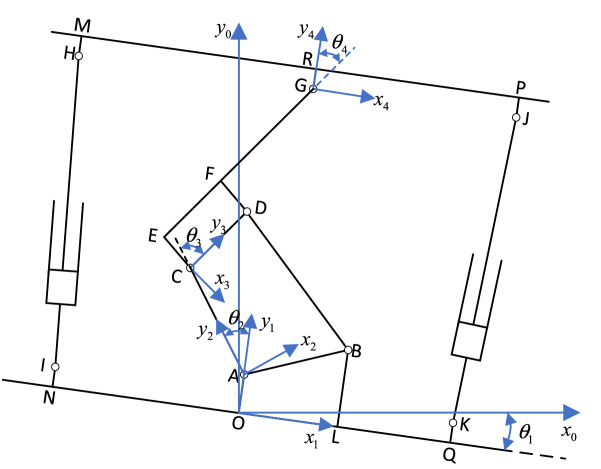
Advanced hydraulic support of the D–H matrix coordinate system.

The coordinate system model based on the D–H matrix is established according to the structural diagram of advanced hydraulic support, as shown in Fig. 8. The key nodes in the figure include A, B, C, D, E, F, G, H, J, M, P, and R. Node A is the hinge point between the base and the rear connecting rod, node B is the hinge point between the base and the front connecting rod, node C is the hinge point between the rear connecting rod and the shield beam, and node D is the hinge point between the front connecting rod and the shield beam. Meanwhile, node G is the hinge point between the shield beam and the top beam, node H is the hinge point between the front column and the top beam, and node J is the hinge point between the rear column and the top beam. Nodes E, F, M, P, and R are the auxiliary calculation nodes set on the edge of each component. Node M and P can represent the support height of the front and rear ends of the advanced hydraulic support.

The hinge joint of each component is selected as the origin coordinate, and the global coordinate system $\left\{ O \right\}$ takes point $O$ as the coordinate origin, the $x$ axis is parallel to the roadway level surface, and the $y$ axis is perpendicular to the roadway level surface. The base coordinate system $\left\{ {O_{{1}} } \right\}$ takes point $O$ as the coordinate origin, the $x_{1}$ axis coincides with the base OL, and the $y_{1}$ axis coincides with OA. The coordinate system of the rear connecting rod $\left\{ {O_{{2}} } \right\}$ is based on point A, the $y_{2}$ axis coincides with AC, the $x_{2}$ axis is perpendicular to AC. The shield beam coordinate system $\left\{ {O_{{3}} } \right\}$ takes point C as the coordinate origin, the $y_{3}$ axis coincides with CD, and the $x_{3}$ axis is perpendicular to CD. The top beam coordinate system $\left\{ {O_{{4}} } \right\}$ takes point G as the coordinate origin, the $x_{4}$ axis is parallel to the top surface of the top beam, and the $y_{4}$ axis coincides with GR.

According to the D–H matrix method, the translation matrix from coordinate system $\left\{ {O_{i} } \right\}$ to $\left\{ {O_{i-1} } \right\}$ is :

1 $$ Trans(t_{x} ,t_{y} ,t_{z} )\left[ { = \begin{array}{*{20}c} 1 & 0 & 0 & {t_{x} } \\ 0 & 1 & 0 & {t_{y} } \\ 0 & 0 & 1 & {t_{z} } \\ 0 & 0 & 0 & 1 \\ \end{array} } \right], $$

where $t_{x}$, $t_{{\text{y}}}$, and $t_{z}$ are the translation quantities of coordinate system $\left\{ {O_{i} } \right\}$ to $\left\{ {O_{i - 1} } \right\}$ on the ${\text{x}}$, $y$, and $z$ axes, respectively.

During lifting and lowering phases, the advance hydraulic support only rotates around the $z$ axis from the coordinate system $\left\{ {O_{i} } \right\}$ to $\left\{ {O_{i - 1} } \right\}$. Thus, the corresponding rotation matrix is as follows:

2 $$ Rotat(z,\theta_{i} ) = \left[ {\begin{array}{*{20}c} {\cos \theta_{i} } & { - \sin \theta_{i} } & 0 & 0 \\ {\sin \theta_{i} } & {\cos \theta_{i} } & 0 & 0 \\ 0 & 0 & 1 & 0 \\ 0 & 0 & 0 & 1 \\ \end{array} } \right] ,$$

where $\theta_{i}$ is the rotation angle of coordinate system $\left\{ {O_{i} } \right\}$ to $\left\{ {O_{i - 1} } \right\}$ around the $z$ axis.

The coordinate transformation matrix of the relative motion from coordinate system $\left\{ {O_{i} } \right\}$ to $\left\{ {O_{i - 1} } \right\}$ obtained by Eqs. (1) and (2) is as follows:

3 $$ {}_{1}^{i - 1} T\quad Trans(t_{x} ,t_{y} ,t_{z} ) \cdot Rotat(y,\varphi_{i} ) = \left[ {\begin{array}{*{20}c} {cos\theta_{1} } & { - sin\theta_{1} } & 0 & {t_{x} } \\ {sin\theta_{1} } & {cos\theta_{1} } & 0 & {t_{y} } \\ 0 & 0 & 1 & {t_{z} } \\ 0 & 0 & 0 & 1 \\ \end{array} } \right] .$$

Coordinate systems $\left\{ {O_{i} } \right\}$ to $\left\{ O \right\}$ can be regarded as the coordinate system $\left\{ {O_{{1}} } \right\}$ rotates the $\theta_{1}$ angle around the $z$ axis $\theta_{1}$ to obtain coordinate system $\left\{ O \right\}$, whose transformation matrix is determined as follows:

4 $$ {}_{1}^{0} T = \left[ {\begin{array}{*{20}c} {cos\theta_{1} } & { - sin\theta_{1} } & 0 & 0 \\ {sin\theta_{1} } & {cos\theta_{1} } & 0 & 0 \\ 0 & 0 & 1 & 0 \\ 0 & 0 & 0 & 1 \\ \end{array} } \right] .$$

Coordinate systems $\left\{ {O_{{2}} } \right\}$ to $\left\{ {O_{{1}} } \right\}$ can be regarded as the coordinate system $\left\{ {O_{{2}} } \right\}$ rotating around the $z$ axis $\theta_{2}$ angles and translating $l_{AO}$ along the negative direction of the $y$ axis to obtain the coordinate system $\left\{ {O_{{1}} } \right\}$, and its transformation matrix is expressed as follows:

5 $$ {}_{2}^{1} T = \left[ {\begin{array}{*{20}c} {cos\theta_{2} } & { - sin\theta_{2} } & 0 & 0 \\ {sin\theta_{2} } & {cos\theta_{2} } & 0 & {l_{AO} } \\ 0 & 0 & 1 & 0 \\ 0 & 0 & 0 & 1 \\ \end{array} } \right]. $$

The coordinate system from $\left\{ {O_{{3}} } \right\}$ to $\left\{ {O_{{2}} } \right\}$ can be regarded as the coordinate system $\left\{ {O_{{3}} } \right\}$ rotates around $z$ axis $\theta_{3}$ angle and translating $l_{AC}$ along the negative direction of the $y$ axis to obtain the coordinate system $\left\{ {O_{{2}} } \right\}$. The transformation matrix is expressed as follows:

6 $$ {}_{3}^{2} T = \left[ {\begin{array}{*{20}c} {cos\theta_{3} } & { - sin\theta_{3} } & 0 & 0 \\ {sin\theta_{3} } & {cos\theta_{3} } & 0 & {l_{AC} } \\ 0 & 0 & 1 & 0 \\ 0 & 0 & 0 & 1 \\ \end{array} } \right] .$$

From the coordinate system $\left\{ {O_{{4}} } \right\}$ to $\left\{ {O_{{3}} } \right\}$, coordinate system $\left\{ {O_{{4}} } \right\}$ rotates around the $z$ axis $\theta_{4}$ angle. First, it moves $l_{GE}$ along the negative direction of the $y$ axis and then $l_{CE}$ along the positive direction of the ${\text{x}}$ axis to obtain coordinate system $\left\{ {O_{{3}} } \right\}$. The transformation matrix is presented as follows:

7 $$ {}_{4}^{3} T = \left[ {\begin{array}{*{20}c} {cos\theta_{4} } & { - sin\theta_{4} } & 0 & {l_{CE} } \\ {sin\theta_{4} } & {cos\theta_{4} } & 0 & {l_{GE} } \\ 0 & 0 & 1 & 0 \\ 0 & 0 & 0 & 1 \\ \end{array} } \right] .$$

The coordinates of any node of the advanced hydraulic support at $\left\{ {O_{i} } \right\}$ can be converted to $\left\{ O \right\}$ in combination with Eqs. (3)–(7) and can be solved by the following transformation relationship:

8 $$ {}_{{\text{i}}}^{0} T = {}_{{1}}^{0} T{}_{{2}}^{1} T{}_{{3}}^{2} T \cdots {}_{{\text{i}}}^{i - 1} T .$$

The transformation matrix from each coordinate system to the global coordinate system can be solved by using Eq. (8) :

9 $$ {}_{1}^{0} T = \left[ {\begin{array}{*{20}c} {cos\theta_{1} } & { - sin\theta_{1} } & 0 & 0 \\ {sin\theta_{1} } & {cos\theta_{1} } & 0 & 0 \\ 0 & 0 & 1 & 0 \\ 0 & 0 & 0 & 1 \\ \end{array} } \right] ,$$

10 $$ {}_{2}^{0} T = \left[ {\begin{array}{*{20}c} {\cos (\theta_{1} + \theta_{2} )} & { - \sin (\theta_{1} + \theta_{2} )} & 0 & { - l_{AO} \sin \theta_{1} } \\ {\sin (\theta_{1} + \theta_{2} )} & {\cos (\theta_{1} + \theta_{2} )} & 0 & {l_{AO} \cos \theta_{1} } \\ 0 & 0 & 1 & 0 \\ 0 & 0 & 0 & 1 \\ \end{array} } \right] ,$$

11 $$ {}_{3}^{0} T = \left[ {\begin{array}{*{20}c} {\cos (\theta_{1} + \theta_{2} + \theta_{3} )} & { - \sin (\theta_{1} + \theta_{2} + \theta_{3} )} & 0 & { - l_{AC} \sin (\theta_{1} + \theta_{2} ) - l_{AO} \sin \theta_{1} } \\ {\sin (\theta_{1} + \theta_{2} + \theta_{3} )} & {\cos (\theta_{1} + \theta_{2} + \theta_{3} )} & 0 & {l_{AC} \cos (\theta_{1} + \theta_{2} ) + l_{AO} \cos \theta } \\ 0 & 0 & 1 & 0 \\ 0 & 0 & 0 & 1 \\ \end{array} } \right], $$

12 $$ \begin{gathered} {}_{4}^{0} T = \left[ {\begin{array}{*{20}c} {\sin (\theta_{1} + \theta_{2} + \theta_{3} + \theta_{4} )} & { - \sin (\theta_{1} + \theta_{2} + \theta_{3} + \theta_{4} )} & 0 \\ {\sin (\theta_{1} + \theta_{2} + \theta_{3} + \theta_{4} )} & {\cos (\theta_{1} + \theta_{2} + \theta_{3} + \theta_{4} )} & 0 \\ 0 & 0 & 1 \\ 0 & 0 & 0 \\ \end{array} } \right. \hfill \\ \left. {\begin{array}{*{20}c} {l_{CE} \cos (\theta_{1} + \theta_{2} + \theta_{3} ) - l_{GE} \sin (\theta_{1} + \theta_{2} + \theta_{3} ) - l_{AC} \sin (\theta_{1} + \theta_{2} ) - l_{AO} \sin \theta_{1} } \\ {l_{CE} \sin (\theta_{1} + \theta_{2} + \theta_{3} ) + l_{GE} \cos (\theta_{1} + \theta_{2} + \theta_{3} ) + l_{AC} \cos (\theta_{1} + \theta_{2} ) + l_{AO} \cos \theta_{1} } \\ 0 \\ 1 \\ \end{array} } \right] \hfill \\ \end{gathered} ,$$

13 $$ \left\{ {\begin{array}{*{20}c} {\theta_{2} = \arctan \frac{{y_{C1} - y_{A1} }}{{x_{{{\text{A}}1}} - x_{C1} }}} \\ {\theta_{3} = \arctan \frac{{x_{D2} - x_{C2} }}{{y_{D2} - y_{C2} }}} \\ {\theta_{4} = \arctan \frac{{x_{R3} - x_{{{\text{G}}3}} }}{{{\text{y}}_{R3} - {\text{y}}_{{{\text{G}}3}} }}} \\ \end{array} } \right. .$$

Based on the transformation relationship between the adjacent coordinate systems of the advanced hydraulic support, the coordinate values of any point on the advanced hydraulic support in the global coordinate system $\left\{ O \right\}$ can be solved.

The coordinates of node $B$ in the $\left\{ {O_{{1}} } \right\}$ coordinate system are shown in Table 1:

**Table 1:** Coordinates of node $B$ in the $\left\{ {O_{{1}} } \right\}$ coordinate system.

Key nodes of the advanced hydraulic support	Coordinates in the $\left\{ {O_{{1}} } \right\}$ coordinate system
$B$	$(l_{OL} ,l_{BL} ,0)$

The coordinates of node $B$ in the $\left\{ O \right\}$ coordinate system are obtained by multiplying Eq. (9) with the coordinates of point $B$ in Table 1 and are shown in Table 2.

**Table 2:** Coordinates of node $B$ in the $\left\{ O \right\}$ coordinate system.

Key nodes of the advanced hydraulic support	Coordinates in the $\left\{ O \right\}$ coordinate system
$B$	$(l_{OL} \cos \theta_{1} - l_{BL} \sin \theta_{1} ,l_{OL} \sin \theta_{1} { + }l_{BL} \cos \theta_{1} ,0)$

The coordinates of node $A$ in the $\left\{ {O_{{2}} } \right\}$ coordinate system are shown in Table 3.

**Table 3:** Coordinates of node $A$ in the $\left\{ {O_{{2}} } \right\}$ coordinate system.

Key nodes of the advanced hydraulic support	Coordinates in the $\left\{ {O_{{2}} } \right\}$ coordinate system
$A$	$(0,\, 0, \, 0)$

The coordinates of node $A$ in the $\left\{ O \right\}$ coordinate system is obtained by multiplying Eq. (10) with the coordinates of point $A$ in Table 3 and are shown in Table 4.

**Table 4:** Coordinates of $A$ in the $\left\{ O \right\}$ coordinate system.

Key nodes of the advanced hydraulic support	Coordinates in the $\left\{ O \right\}$ coordinate system
$A$	$( - L_{AO} \sin \theta_{1} ,L_{AO} \cos \theta_{1} ,0)$

The coordinates of nodes $C$, $D$, $E$, and $F$ in the $\left\{ {O_{{3}} } \right\}$ coordinate system are shown in Table 5.

**Table 5:** Coordinates of nodes $C$, $D$, $E$, and $F$ in the $\left\{ {O_{{3}} } \right\}$ coordinate system.

Key nodes of the advanced hydraulic support	Coordinates in the $\left\{ {O_{{3}} } \right\}$ coordinate system
$C$	$(0,\;0,\;0)$
$D$	$(0,l_{CD} ,0)$
$E$	$( - l_{CE} ,0,0)$
$F$	$( - l_{CE} ,l_{EF} ,0)$

The coordinates of nodes $C$, $D$, $E$, and $F$ are obtained in the $\left\{ O \right\}$ coordinate system can be obtained by multiplying Eq. (11) with the coordinates of each point in Table 5 and shown in Table 6.

**Table 6:** Coordinates of nodes $C$, $D$, $E$, and $F$ in the $\left\{ O \right\}$ coordinate system.

Key nodes of the advanced hydraulic support	Coordinates in the $\left\{ O \right\}$ coordinate system
$C$	$( - l_{AC} \sin (\theta_{1} + \theta_{2} ) - l_{AO} \sin \theta_{1} ,l_{AC} \cos (\theta_{1} + \theta_{2} ) + l_{AO} \cos \theta_{1} ,0)$
$D$	$$\begin{gathered} ( - l_{CD} \sin (\theta_{1} + \theta_{2} + \theta_{3} ) - l_{AC} \sin (\theta_{1} + \theta_{2} ) - l_{AO} \sin \theta_{1} , \hfill \\ l_{CD} \cos (\theta_{1} + \theta_{2} + \theta_{3} ) + l_{AC} \cos (\theta_{1} + \theta_{2} ) + l_{AO} \cos \theta_{1} ,0) \hfill \\ \end{gathered}$$
$E$	$$\begin{gathered} ( - l_{CE} \cos (\theta_{1} + \theta_{2} + \theta_{3} ) - l_{AC} \sin (\theta_{1} + \theta_{2} ) - l_{AO} \sin \theta_{1} , \hfill \\ - l_{CE} \sin (\theta_{1} + \theta_{2} + \theta_{3} ) + l_{AC} \cos (\theta_{1} + \theta_{2} ) + l_{AO} \cos \theta_{1} ,0) \hfill \\ \end{gathered}$$
$F$	$$\begin{gathered} ( - l_{CE} \cos (\theta_{1} + \theta_{2} + \theta_{3} ) - l_{EF} \sin (\theta_{1} + \theta_{2} + \theta_{3} ) - l_{AC} \sin (\theta_{1} + \theta_{2} ) - l_{AO} \sin \theta_{1} , \hfill \\ - l_{CE} \sin (\theta_{1} + \theta_{2} + \theta_{3} ) + l_{EF} \cos (\theta_{1} + \theta_{2} + \theta_{3} ) + l_{AC} \cos (\theta_{1} + \theta_{2} ) + l_{AO} \cos \theta_{1} ,0) \hfill \\ \end{gathered}$$

The coordinates of nodes $G$, $H$, $J$, $M$, $P$, and $R$ in the $\left\{ {O_{{4}} } \right\}$ coordinate system are shown in Table 7.

**Table 7:** Coordinates of nodes $G$, $H$, $J$, $M$, $P$, and $R$ in the $\left\{ {O_{{4}} } \right\}$ coordinate system.

Key nodes of the advanced hydraulic support	Coordinates in the $\left\{ {O_{{4}} } \right\}$ coordinate system
$G$	$(0,\, 0, \, 0)$
$H$	$( - l_{MR} ,0,0)$
$J$	$(l_{PR} ,0,0)$
$M$	$( - l_{{{\text{MR}}}} ,l_{GR} ,0)$
$P$	$(l_{{{\text{PR}}}} ,l_{GR} ,0)$
$R$	$(0,l_{GR} ,0)$

Equation (14) is multiplied by the coordinates of each point in Table 7, and the coordinates of nodes $G$, $H$, $J$, $M$, $P$, and $R$ in the $\left\{ O \right\}$ coordinate system are obtained, as shown in Table 8.

**Table 8:** Coordinates of nodes $G$, $H$, $J$, $M$, $P$, and $R$ in the $\left\{ O \right\}$ coordinate system.

Key nodes of the advanced hydraulic support	Coordinates in the $\left\{ O \right\}$ coordinate system
$G$	$$\begin{gathered} (l_{CE} \cos (\theta_{1} + \theta_{2} + \theta_{3} ) + l_{GE} \sin (\theta_{1} + \theta_{2} + \theta_{3} ) - l_{AC} \sin (\theta_{1} + \theta_{2} ) - l_{AO} \sin \theta_{1} , \hfill \\ l_{CE} \sin (\theta_{1} + \theta_{2} + \theta_{3} ) + l_{GE} \cos (\theta_{1} + \theta_{2} + \theta_{3} ) + l_{AC} \cos (\theta_{1} + \theta_{2} ) + l_{AO} \cos \theta_{1} ,0) \hfill \\ \end{gathered}$$
$H$	$$\begin{gathered} ( - l_{MR} \cos (\theta_{1} + \theta_{2} + \theta_{3} + \theta_{4} ) + l_{CE} \cos (\theta_{1} + \theta_{2} + \theta_{3} ) - l_{GE} \sin (\theta_{1} + \theta_{2} + \theta_{3} ) \hfill \\ - l_{AC} \sin (\theta_{1} + \theta_{2} ) - l_{AO} \sin \theta_{1} , - l_{MR} \sin (\theta_{1} + \theta_{2} + \theta_{3} + \theta_{4} ) + l_{CE} \sin (\theta_{1} + \theta_{2} \hfill \\ + \theta_{3} ) + l_{GE} \cos (\theta_{1} + \theta_{2} + \theta_{3} ) + l_{AC} \cos (\theta_{1} + \theta_{2} ) + l_{AO} \cos \theta_{1} ,0) \hfill \\ \end{gathered}$$
$J$	$$\begin{gathered} (l_{PR} \cos (\theta_{1} + \theta_{2} + \theta_{3} + \theta_{4} ) + l_{CE} \cos (\theta_{1} + \theta_{2} + \theta_{3} ) - l_{GE} \sin (\theta_{1} + \theta_{2} + \theta_{3} ) \hfill \\ - l_{AC} \sin (\theta_{1} + \theta_{2} ) - l_{AO} \sin \theta_{1} ,l_{PR} \sin (\theta_{1} + \theta_{2} + \theta_{3} + \theta_{4} ) + l_{CE} \sin (\theta_{1} + \theta_{2} + \theta_{3} ) \hfill \\ + l_{GE} \cos (\theta_{1} + \theta_{2} + \theta_{3} ) + l_{AC} \cos (\theta_{1} + \theta_{2} ) + l_{AO} \cos \theta_{1} ,0) \hfill \\ \end{gathered}$$
$M$	$$\begin{gathered} ( - l_{MR} \cos (\theta_{1} + \theta_{2} + \theta_{3} + \theta_{4} ) - l_{GR} \sin (\theta_{1} + \theta_{2} + \theta_{3} + \theta_{4} ) + l_{CE} \cos (\theta_{1} + \theta_{2} + \theta_{3} ) \hfill \\ - l_{GE} \sin (\theta_{1} + \theta_{2} + \theta_{3} ) - l_{AC} \sin (\theta_{1} + \theta_{2} ) - l_{AO} \sin \theta_{1} , - l_{{{\text{MR}}}} \sin (\theta_{1} + \theta_{2} + \theta_{3} + \theta_{4} ) \hfill \\ + l_{GR} \cos (\theta_{1} + \theta_{2} + \theta_{3} + \theta_{4} ) + l_{CE} \sin (\theta_{1} + \theta_{2} + \theta_{3} ) + l_{GE} \cos (\theta_{1} + \theta_{2} + \theta_{3} ) \hfill \\ + l_{AC} \cos (\theta_{1} + \theta_{2} ) + l_{AO} \cos \theta_{1} ,0) \hfill \\ \end{gathered}$$
$P$	$$\begin{gathered} (l_{{{\text{PR}}}} \cos (\theta_{1} + \theta_{2} + \theta_{3} + \theta_{4} ) - l_{GR} \sin (\theta_{1} + \theta_{2} + \theta_{3} + \theta_{4} ) + l_{CE} \cos (\theta_{1} + \theta_{2} + \theta_{3} ) \hfill \\ - l_{GE} \sin (\theta_{1} + \theta_{2} + \theta_{3} ) - l_{AC} \sin (\theta_{1} + \theta_{2} ) - l_{AO} \sin \theta_{1} ,l_{{{\text{PR}}}} \sin (\theta_{1} + \theta_{2} + \theta_{3} + \theta_{4} ) \hfill \\ + l_{GR} \cos (\theta_{1} + \theta_{2} + \theta_{3} + \theta_{4} ) + l_{CE} \sin (\theta_{1} + \theta_{2} + \theta_{3} ) + l_{GE} \cos (\theta_{1} + \theta_{2} + \theta_{3} ) \hfill \\ + l_{AC} \cos (\theta_{1} + \theta_{2} ) + l_{AO} \cos \theta_{1} ,0) \hfill \\ \end{gathered}$$
$R$	$$\begin{gathered} ( - l_{GR} \sin (\theta_{1} + \theta_{2} + \theta_{3} + \theta_{4} ) + l_{CE} \cos (\theta_{1} + \theta_{2} + \theta_{3} ) - l_{GE} \sin (\theta_{1} + \theta_{2} + \theta_{3} ) \hfill \\ - l_{AC} \sin (\theta_{1} + \theta_{2} ) - l_{AO} \sin \theta ,l_{GR} \cos (\theta_{1} + \theta_{2} + \theta_{3} + \theta_{4} ) + l_{CE} \sin (\theta_{1} + \theta_{2} + \theta_{3} ) \hfill \\ + l_{GE} \cos (\theta_{1} + \theta_{2} + \theta_{3} ) + l_{AC} \cos (\theta_{1} + \theta_{2} ) + l_{AO} \cos \theta_{1} ,0) \hfill \\ \end{gathered}$$

In the process of lifting and falling of a single group of advanced hydraulic support, each actuator is regarded as only rotating around the $z$ axis. The pitch angle $\varphi$ of the base, the pitch angle $\eta$ of the top beam, and the pitch angle $\beta$ of the rear connecting rod are obtained with the attitude sensor. During the working process, point B will change. The changing $l_{BC}$ length is solved with the help of the known parameters of the actuator and the conversion relationship between the coordinate systems. Then, angle $\tau$ is obtained by the simultaneous solution of the known attitude parameters of each component and the inverse trigonometric function relationship:

14 $$ \tau = \arccos \frac{{l_{CB}^{2} + l_{CD}^{2} - l_{BD}^{2} }}{{2 \times l_{CB} \times l_{CD} }} - \arccos \frac{{l_{CA}^{2} + l_{CB}^{2} - l_{AB}^{2} }}{{2 \times l_{CB} \times l_{CD} }} .$$

Then, conversion angle $\theta_{i}$ of each coordinate system is obtained by solving:

15 $$ \left\{ {\begin{array}{*{20}l} {\theta_{1} = \varphi } \hfill \\ {\theta_{2} = \beta + \theta_{1} - \frac{\pi }{2}} \hfill \\ {\theta_{3} = \pi - \tau } \hfill \\ {\theta_{4} = \frac{\pi }{2} - \alpha - \eta } \hfill \\ \end{array} } \right. .$$

Based on the above equation, the shield beam pitch angle $\alpha$ is:

16 $$ \alpha = \beta - \theta_{3} .$$

The coordinates of each key node in the global coordinate system are obtained using the above equation. The pose parameters of each actuator of the advanced hydraulic support can be solved together with the length parameters of each actuator.

### Construction of the kinematic model of the advanced hydraulic support group

**Figure 9:**
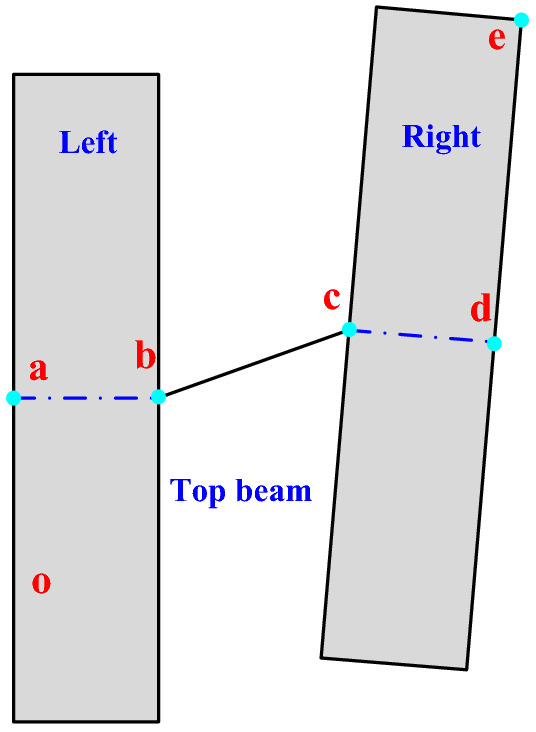
2D model diagram of offset between leading hydraulic supports.

The relative deviation between the advanced hydraulic support groups mainly includes the pitch, roll, and yaw angle deviations. In the actual support conditions, the deviation of the yaw angle between the supports has the greatest influence on the safety support of the advanced hydraulic support. Taking the deviation of the yaw angle between the groups of the advanced hydraulic support as an example, the kinematics between the advanced hydraulic support is analyzed, and the motion model of the advanced hydraulic support group is simplified, as shown in Fig. 9. Based on the horizontal global coordinate system, the kinematics equation is established by combining the D–H matrix and the geometric analysis method, and the pose parameters of key nodes are solved.

**Figure 10:**
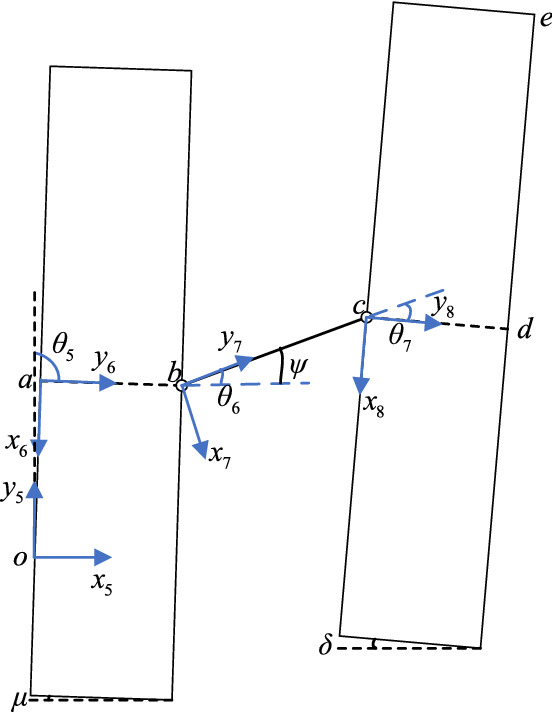
D–H matrix coordinate system between leading hydraulic supports.

A D–H matrix coordinate system is established, as shown in Fig. 10. The hinge joint of each component is selected as the origin coordinate, and the reference coordinate system $\left\{ {o_{5} } \right\}$ takes point $o$ as the origin of the coordinate, and its ${\text{y}}_{5}$ axis coincides with $oa$, which is vertically upward, and the $x_{5}$ axis is perpendicular to $oa$. Coordinate system $\left\{ {o_{6} } \right\}$ of the left advanced hydraulic support takes point $\alpha$ as the origin of the coordinate system, and its $x_{6}$ axis coincides with the base $oa$, vertically downward, and the $y_{6}$ axis is perpendicular to $oa$. Balance cylinder coordinate system $\left\{ {o_{7} } \right\}$ takes point $b$ as coordinate origin, $y_{7}$ axis coincides with balance cylinder *bc*, and the $x_{7}$ axis is perpendicular to *bc*. The coordinate system $\left\{ {o_{8} } \right\}$ of the right advanced hydraulic support is based on point $c$, where the $y_{8}$ axis coincides with the base *cd,* and the $x_{8}$ axis is perpendicular to the *cd*.

Based on the derivation of the coordinate transformation matrix in the previous section, coordinate system $\left\{ {o_{6} } \right\}$ to $\left\{ {o_{5} } \right\}$ can be regarded as the coordinate system $\left\{ {o_{6} } \right\}$ rotates along the $z$ axis $\theta_{5}$ angle and translating $l_{oa}$ along the negative direction of the $y$ axis of the transformed coordinate system to obtain the coordinate system $\left\{ {o_{5} } \right\}$. The transformation matrix is expressed as follows:

17 $$ {}_{6}^{5} T = \left[ {\begin{array}{*{20}c} {cos\theta_{5} } & { - sin\theta_{5} } & 0 & 0 \\ {sin\theta_{5} } & {cos\theta_{5} } & 0 & {l_{{{\text{oa}}}} } \\ 0 & 0 & 1 & 0 \\ 0 & 0 & 0 & 1 \\ \end{array} } \right] .$$

The coordinate system from $\left\{ {o_{7} } \right\}$ to $\left\{ {o_{6} } \right\}$ can be regarded as the coordinate system $\left\{ {o_{7} } \right\}$ rotates the $\theta_{6}$ angle along the $z$ axis and translating $l_{ab}$ along the negative direction of the $y$ axis to obtain coordinate system $\left\{ {o_{6} } \right\}$. The transformation matrix is as follows:

18 $${}_{7}^{6} T = \left[ {\begin{array}{*{20}c} {cos\theta_{6} } & { - sin\theta_{6} } & 0 & 0 \\ {sin\theta_{6} } & {cos\theta_{6} } & 0 & {l_{{{\text{ab}}}} } \\ 0 & 0 & 1 & 0 \\ 0 & 0 & 0 & 1 \\ \end{array} } \right] .$$

The coordinate system from $\left\{ {o_{8} } \right\}$ to $\left\{ {o_{7} } \right\}$ can be regarded as the coordinate system $\left\{ {o_{8} } \right\}$ rotating the $\theta_{7}$ angle along the $z$ axis and translating $l_{bc}$ along the negative direction of the $y$ axis to obtain coordinate system $\left\{ {o_{7} } \right\}$. The transformation matrix is expressed as follows:

19 $${}_{8}^{7} T = \left[ {\begin{array}{*{20}c} {cos\theta_{7} } & { - sin\theta_{7} } & 0 &0 \\ {sin\theta_{7} } &{cos\theta_{7} } & 0 & {l_{{{\text{bc}}}} } \\ 0 & 0 & 1 &0 \\ 0 & 0 & 0 & 1 \\ \end{array} } \right] .$$

The coordinates of any node of the advanced hydraulic support group at $\left\{ {o_{i} } \right\}$ can be converted to $\left\{ o \right\}$ using Eqs. (17)–(19) and can be solved by the following transformation relationship:

20 $$ {}_{{\text{i}}}^{0} T = {}_{{1}}^{0} T{}_{{2}}^{1} T{}_{{3}}^{2} T \cdots {}_{{\text{i}}}^{i - 1} T. $$

The transformation matrix from each coordinate system to the global coordinate system can be solved by using Eq. (20):

21 $$ {}_{6}^{5} T = \left[ {\begin{array}{*{20}c} {cos\theta_{5} } & { - sin\theta_{5} } & 0 & 0 \\ {sin\theta_{5} } & {cos\theta_{5} } & 0 & {l_{{{\text{oa}}}} } \\ 0 & 0 & 1 & 0 \\ 0 & 0 & 0 & 1 \\ \end{array} } \right] ,$$

22 $$ {}_{7}^{5} T = {}_{6}^{5} T{}_{7}^{6} T = \left[ {\begin{array}{*{20}c} {\cos (\theta_{5} + \theta_{6} )} & { - \sin (\theta_{5} + \theta_{6} )} & 0 & { - l_{ab} \sin \theta_{5} } \\ {\sin (\theta_{5} + \theta_{6} )} & {\cos (\theta_{5} + \theta_{6} )} & 0 & {l_{ab} \cos \theta_{5} + l_{oa} } \\ 0 & 0 & 1 & 0 \\ 0 & 0 & 0 & 1 \\ \end{array} } \right] ,$$

23 $$ {}_{8}^{5} T = {}_{6}^{5} T{}_{7}^{6} T{}_{8}^{7} T = \left[ {\begin{array}{*{20}c} {\cos (\theta_{5} + \theta_{6}+  \theta_{7})} & { - \sin (\theta_{5} + \theta_{6}  + \theta_{7})} & 0 & { - l_{bc} \sin (\theta_{5}+\theta_{6} }){ - l_{ab} \sin \theta_{5} } \\ {\sin (\theta_{5} + \theta_{6} +\theta_{7} )} & {\cos (\theta_{5} + \theta_{6} +\theta_{7} )} & 0 & {l_{bc} (\cos \theta_{5} +\theta_{6}}) +{l_{ab} \cos \theta_{5} + l_{oa} }\\ 0 & 0 &1 & 0 \\ 0 & 0 & 0 & 1 \\ \end{array} } \right] ,$$

In the side pushing process of the advanced hydraulic support group, each actuator is regarded as rotating only around the $z$ axis. The yaw angle $\psi$ of the balance cylinder, the yaw angle $\mu$ of the left advanced hydraulic support and the yaw angle $\delta$ of the right advanced hydraulic support are obtained with the attitude sensor. Then, the conversion angle $\theta_{i}$ of each coordinate system is obtained as follows:

24 $$ \left\{ {\begin{array}{*{20}l} {\theta_{5} = \frac{\pi }{2}{ + }\mu } \hfill \\ {\theta_{6} = \psi } \hfill \\ {\theta_{7} = \psi + \delta } \hfill \\ \end{array} } \right. .$$

Based on the transformation relationship between the coordinate systems of the adjacent components of the above advanced hydraulic group, the coordinate values of any point on the advanced hydraulic support in the reference coordinate system $\left\{ {o_{5} } \right\}$ can be solved. The key nodes in the structural sketch of the advanced hydraulic support are $\alpha$, $b$, $c$, $d$, and $e$*.*

The coordinates of node $\alpha$ in the $\left\{ {o_{6} } \right\}$ coordinate system are shown in Table 9.

**Table 9:** Coordinates of node $\alpha$ in the $\left\{ {o_{6} } \right\}$ coordinate system.

Key nodes of the advanced hydraulic support	Coordinates in the $\left\{ {o_{6} } \right\}$ coordinate system
$\alpha$	$(0,\, 0, \, 0)$

Equation (21) is multiplied by the coordinates of the $\alpha$ point in Table 9 to obtain the coordinates of *a* in the $\left\{ {o_{5} } \right\}$ coordinate system, as shown in Table 10.

**Table 10:** Coordinates of node $\alpha$ in the $\left\{ {o_{5} } \right\}$ coordinate system.

Key nodes of the advanced hydraulic support	Coordinates in the $\left\{ {o_{5} } \right\}$ coordinate system
$\alpha$	$(0,l_{oa} ,0)$

The coordinates of node $b$ in the $\left\{ {o_{7} } \right\}$ coordinate system are easily obtained, as shown in Table 11.

**Table 11:** Coordinates of node $b$ in the $\left\{ {o_{7} } \right\}$ coordinate system.

Key nodes of the advanced hydraulic support	Coordinates in the $\left\{ {o_{7} } \right\}$ coordinate system
$b$	$(0,\, 0, \, 0)$

The coordinates of point $b$ in the $\left\{ {o_{5} } \right\}$ coordinate system are obtained by multiplying Eq. (22) with the coordinates of point $b$ in Table 11, as shown in Table 12.

**Table 12:** Coordinates of node $b$ in the $\left\{ {o_{5} } \right\}$ coordinate system.

Key nodes of the advanced hydraulic support	Coordinates in the $\left\{ {o_{5} } \right\}$ coordinate system
$b$	$( - l_{ab} \sin \theta_{5} ,l_{ab} \cos \theta_{5} + l_{oa} ,0)$

The coordinates of nodes $c$, $d$, and $e$ in the $\left\{ {o_{8} } \right\}$ coordinate system are shown in Table 13.

**Table 13:** Coordinates of nodes $c$, $d$, and $e$ in the $\left\{ {o_{8} } \right\}$ coordinate system.

Key nodes of the advanced hydraulic support	Coordinates in the $\left\{ {o_{8} } \right\}$ coordinate system
$c$	$(0,\, 0, \, 0)$
$d$	$(0,l_{cd} ,0)$
$e$	$( - l_{de} , - l_{cd} ,0)$

The coordinates of nodes $c$, $d$, and $e$ in the $\left\{ {o_{5} } \right\}$ coordinate system are obtained by multiplying Eq. (23) with the coordinates of each point in Table 13, as shown in Table 14.

**Table 14:** Coordinates of nodes $c$, $d$, and $e$ in the $\left\{ {o_{5} } \right\}$ coordinate system.

Key nodes of the advanced hydraulic support	Coordinates in the $\left\{ {o_{5} } \right\}$ coordinate system
$c$	$( - l_{bc} \sin (\theta_{5} + \theta_{6} ) - l_{ab} \sin \theta_{5} ,l_{bc} \cos (\theta_{5} + \theta_{6} ) + l_{ab} \cos \theta_{5} + l_{oa} ,0)$
$d$	$$\begin{gathered} ( - l_{cd} sin(\theta_{5} + \theta_{6} + \theta_{7} ) - l_{bc} sin(\theta_{5} + \theta_{6} ) - l_{ab} sin\theta_{5} , \hfill \\ l_{cd} cos(\theta_{5} + \theta_{6} + \theta_{7} ) + l_{bc} cos(\theta_{5} + \theta_{6} ) + l_{ab} cos\theta_{5} + l_{oa} ,0) \hfill \\ \end{gathered}$$
$e$	$$\begin{gathered} ( - l_{de} cos(\theta_{5} + \theta_{6} + \theta_{7} ) - l_{cd} sin(\theta_{5} + \theta_{6} + \theta_{7} ) - l_{bc} sin(\theta_{5} + \theta_{6} ) - l_{ab} sin\theta_{5} , \hfill \\ - l_{de} sin(\theta_{5} + \theta_{6} + \theta_{7} ) + l_{cd} cos(\theta_{5} + \theta_{6} + \theta_{7} ) + l_{bc} cos(\theta_{5} + \theta_{6} ) + l_{ab} cos\theta_{5} + l_{oa} ,0) \hfill \\ \end{gathered}$$

## Kinematic simulation analysis of the advanced hydraulic support group

### Kinematic simulation analysis of the single-group advanced hydraulic support

**Figure 11:**
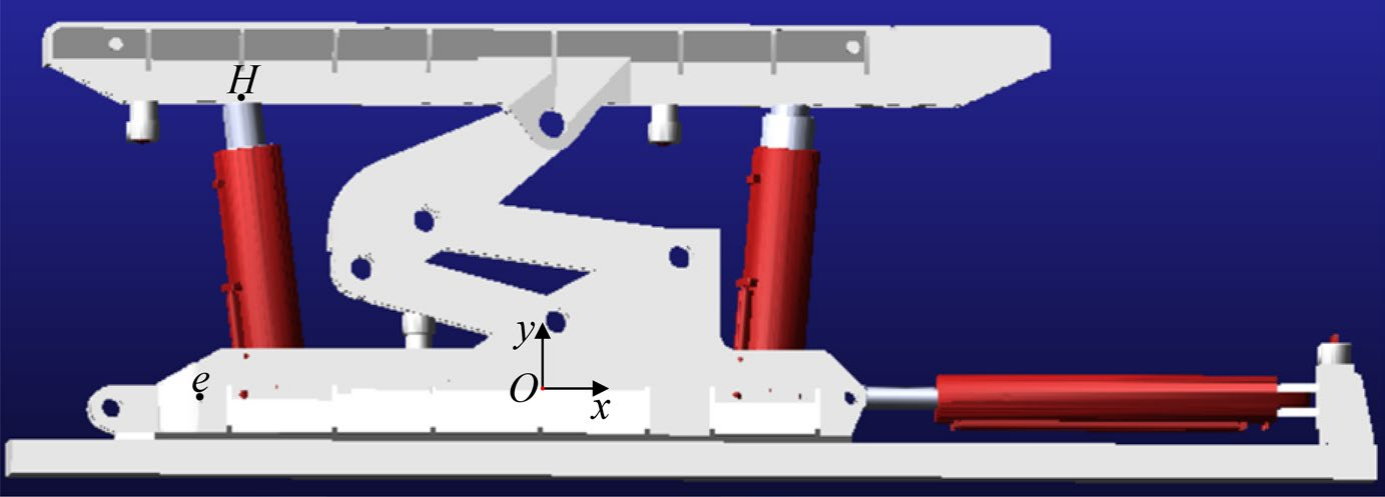
Kinematic simulation model diagram of the advanced hydraulic support.

The kinematic simulation software ADAMS is used to create a single group of advanced hydraulic support simulation model, and the kinematic simulation experiment is carried out. The accuracy and effectiveness of the kinematics model are verified, as shown in Fig. 11. The software is mainly composed of the simulation model of the advanced hydraulic support and the simulation floor of the roadway. The base and the floor and the floor and the ground are connected by a fixed pair. The actuators, such as the column, the balance cylinder, and the push cylinder are connected by a sliding pair and added a sliding drive. The shield beam, the front connecting rod, and the rear connecting rod are connected by a rotating pair. In the figure, point $O$ is the origin of the global coordinate system, the $y$ axis is perpendicular to the floor, the $x$ axis is parallel to the floor, point $H$ is the monitoring point of the support height, and point $e$ is the relative position monitoring point of the advanced hydraulic support group. The monitoring points are added to the sliding and rotating pairs to monitor the angle change of the actuator and the change of the telescopic length in the support process.

The simulation model is imported into ADAMS software, and the quality attributes, gravity direction, and other parameters of each mechanism are set up. The constraint relationship and driving mode between the actuators are established. Furthermore, the corresponding coordinate system and monitoring points are established in the simulation model according to the established pose model coordinate system, and the basic setting of the simulation model is completed.

The key dimensions of the model are shown in Table 15.

**Table 15:** Structural parameters of the key components of the simulation model.

Key components of the simulation model	Parameter value (mm)	Key components of the simulation model	Parameter value (mm)
$l_{AO}$	390	$l_{GJ}$	235
$l_{AC}$	590	$l_{GR}$	389
$l_{AB}$	315	$l_{{{\text{oa}}}}$	570
$l_{BD}$	640	$l_{{{\text{ab}}}}$	200
$l_{CD}$	505	$l_{{{\text{cd}}}}$	200
$l_{GE}$	930	$l_{{{\text{de}}}}$	340
$l_{HG}$	270		

Taking the inclination level of the base of the advanced hydraulic support as an example, the monitoring points are added at the front end of the top beam, the shield beam, and the rear connecting rod to more intuitively understand the support height of advanced hydraulic support and the attitude angle of each actuator during the movement of advanced hydraulic support. The *Step* function was used to set the driving modes of the four columns. The simulation time was set to 5 s, and the simulation step was set to 200. Accordingly, the four columns were increased by 510 mm within 0–5 s. During this process, the front support height $H$ of the top beam, the inclination angle $\alpha$ of the rear connecting rod, and the inclination angle $\beta$ of the shield beam were obtained.

The kinematic simulation results are shown in Table 16.

**Table 16:** Kinematic simulation results of the advanced hydraulic support.

Support height $H$ (mm)	Dip angle of back link $\alpha$ (mm)	Angle of shield beam $\beta$ (mm)
990	40.39	165.03
993	40.48	164.82
996	40.58	164.61
998	40.64	164.47
$\cdots$	$\cdots$	$\cdots$
1498	61.42	124.03
1500	61.56	123.83

**Figure 12:**
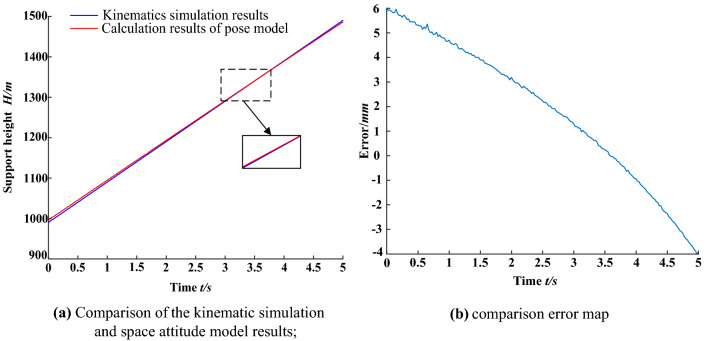
Comparison of the simulation results of the kinematic support height of the advance hydraulic support.

The data sets of the base angle and rear connecting rod angle obtained by kinematic simulation are imported into the MATLAB program of the spatial posture model of the advanced hydraulic support. The calculation results of the posture model of the advanced hydraulic support are compared with the kinematic simulation results. The comparison of the front support height $H$ of the top beam is shown in Fig. 12.

In Fig. 12, the calculation results of the advanced hydraulic support pose model are basically consistent with the kinematic simulation results when the base of the advanced hydraulic support is in the horizontal state. The calculated value coincides with the simulated value in the range of 3–4 s. The error of the support height is less than ± 6 mm, and the average absolute error is 2.92 mm throughout the lifting process. The established advanced hydraulic support space attitude model can accurately feedback the advanced hydraulic space attitude information.

### Kinematic simulation analysis of the advanced hydraulic support group

The monitoring points were added at the hinge joint between the balance cylinder and the base and the front end of the right base to more intuitively understand the relative position motion relationship of the advanced hydraulic support group based on the left advanced hydraulic support. The step function is used to set the driving mode of the balance cylinder ahead of the top beam. The simulation time is set to 5 s, and the simulation step length is set to 50. Accordingly, the balance cylinder ahead of the top beam is pushed sideways by 240 mm in 0–5 s. The (*x*,*y*) coordinate transformation relationship between the top beam expansion length $L$ of the base balance cylinder, the deflection angle $\psi$ of the balance cylinder, the yaw angle $\delta$ of the right advanced hydraulic support, and the monitoring point $e$ of the right advanced hydraulic support is obtained. The kinematic simulation results are shown in Table 17.

**Table 17:** Kinematic simulation results of the advanced hydraulic support group.

Balance cylinder stretching length $L$ (mm)	Balance cylinder deflection angle $\psi$ (mm)	Right bracket yaw angle $\delta$ (mm)	*e* point coordinates (*x*, *y*) (mm)
948.38017	0	0	(1344.03902, 901.55408)
950.54249	0.01453	0.21744	(1348.15444, 900.77253)
952.70041	0.01898	0.41339	(1352.26711, 899.97987)
954.85979	0.02342	0.61591	(1356.37806, 899.18072)
957.01977	0.02794	0.82143	(1360.48727, 898.37302)
$\cdots$	$\cdots$	$\cdots$	$\cdots$
1052.98873	0.35781	9.57326	(1539.10207, 853.52522)
1055.19956	0.36824	9.90811	(1543.11251, 852.29452)
1057.41199	0.37879	10.48845	(1547.12169, 851.05436)

**Figure 13:**
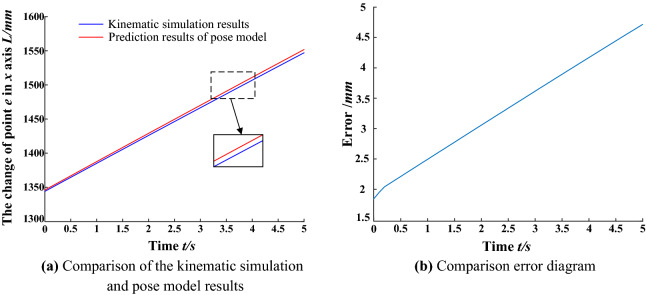
Comparison of the kinematic simulation results of the x-axis position change of the monitoring points.

**Figure 14:**
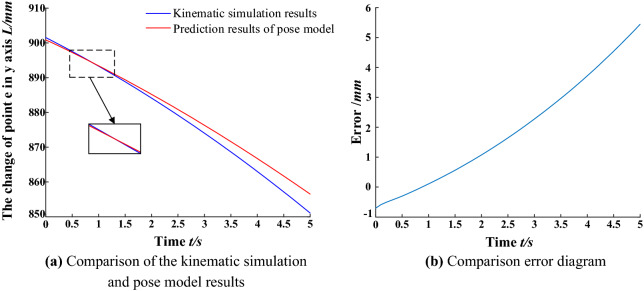
Comparison of the kinematic simulation results of the monitoring point y-axis variation.

The data sets, such as the telescopic length of the balanced cylinder and the deflection angle of the balanced cylinder obtained by kinematic simulation, are imported into the MATLAB program of the spatial attitude model between the advanced hydraulic support groups. The calculation results of the spatial attitude model of the advanced hydraulic support group are compared with the kinematic simulation results. The coordinate (*x*,*y*) transformation of monitoring point *e* is shown in Figs. 13 and 14.

In the aforementioned figures, the calculation results of the pose model between the advanced hydraulic support are basically consistent with the kinematic simulation results when the right advanced hydraulic support is moved to the right with the left advanced hydraulic support as the reference support. In the range of 0.5–1.5 s, the $y$ axis simulation and calculation values overlap without deviation. Meanwhile, the $x$ axis position simulation and calculation value deviation is the largest in the range of 3–4 s and in the controllable range. The error of the measurement results of the $e$ point (*x*,*y*) is within ± 6 mm. The average absolute error of the $x$ axis is 3.26 mm, and that of the $y$ axis is 1.99 mm, which meet the requirements of the pose monitoring accuracy of the advanced hydraulic support. The correctness and effectiveness of the spatial pose model of the advanced hydraulic support group are verified by the comparative analysis of the results. The support height of the advanced hydraulic support and the relative pose information between the supports can be accurately calculated by the model, which provides a theoretical basis for the pose monitoring scheme of the advanced hydraulic support.

## Experimental verification analysis

**Figure 15:**
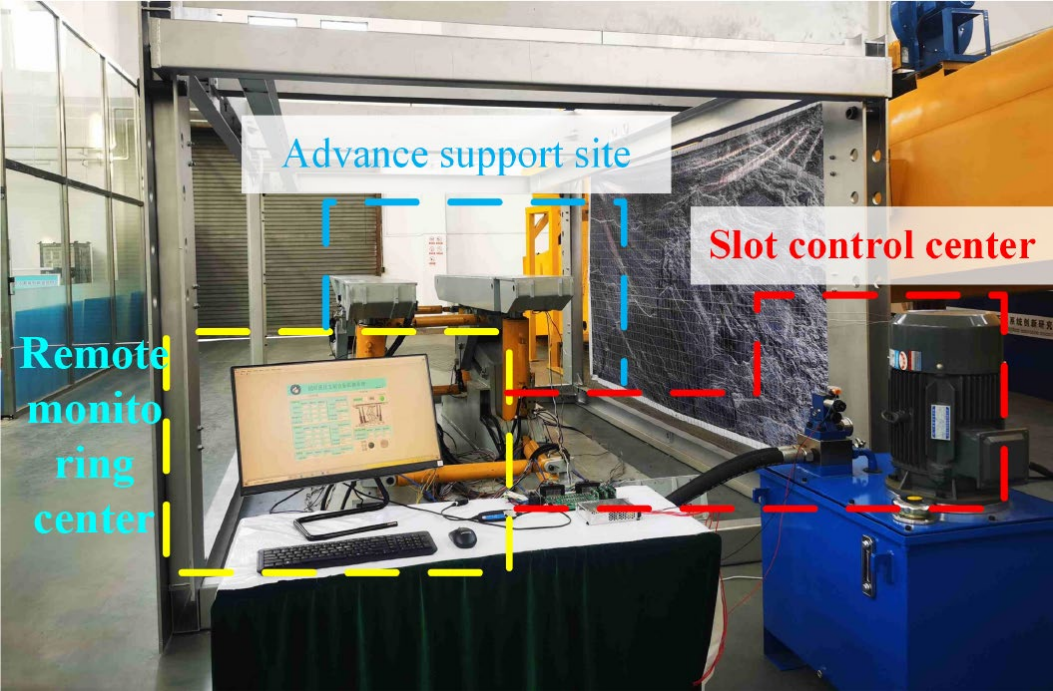
Overall structure of the advanced hydraulic support experimental system.

The advanced hydraulic support attitude test experimental platform built by Shandong University of Science and Technology Robot Center is designed according to the simulation and actual scaling ratio of 1:2, as shown in Fig. 15. The platform can simulate the actual working conditions of the advanced hydraulic support group in the roadway and complete the lifting, falling, and moving of the advanced hydraulic support group. Moreover, the platform is divided into advanced support site, control center, and remote monitoring center.

The main technical parameters of the advanced hydraulic support posture monitoring experimental platform are shown in Table 18.

**Table 18:** Main technical parameters of the advanced hydraulic support experimental prototype.

Parameter name	Numerical value
Maximum support height of the prototype	2 m
Minimum height of the prototype	1 m
Support width of prototype	2 m
Width of the experimental platform	3 m
Length of the experimental platform	4.5 m
Working pressure of the system	31.5 MPa
Motor power of the system	37 kW

**Figure 16:**
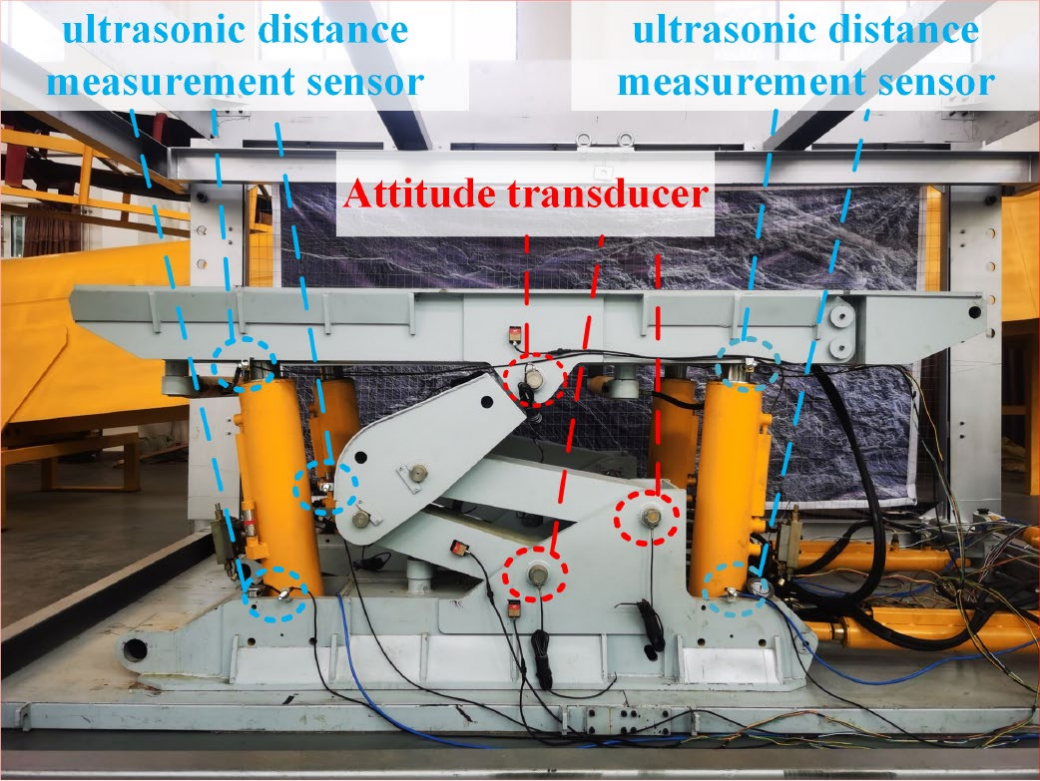
Installation layout of the pose monitoring module.

In the experiment, the ultrasonic ranging sensor with model US1000-G18-U-M12.1, the inclination sensor with model HWT905-CAN, and the stroke sensor built in the cylinder were used to measure the overall attitude of the advanced hydraulic support group. The inclination sensor is installed on the top beam, the base, and the balance cylinder of the support to collect the attitude information of the corresponding position. The ultrasonic ranging sensor is installed at the two ends of the top beam, the two ends of the base, and the lower end of the shield beam to collect the distance between the support and the two sides of the roadway. The cylinder stroke sensor uses an internal sensor to collect the telescopic length of the cylinder. The surface of the tilt sensor should be attached to the support surface as far as possible to avoid gaps. The installation direction of the sensor is consistent with the direction expressed in the pose monitoring model. The specific placement of the pose monitoring sensor is shown in Fig. 16.

**Figure 17:**
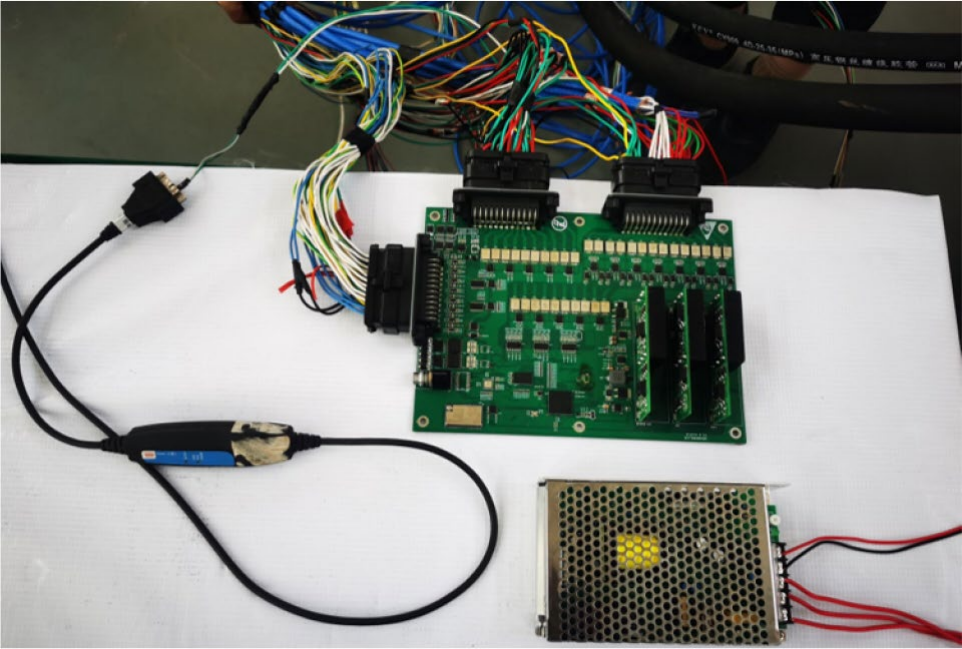
Controller of the advanced hydraulic support group.

The ultrasonic ranging sensor and the cylinder stroke sensor used in the experiment are both analog output sensors. Accordingly, the advanced hydraulic support controller is independently developed using the ADC circuit to convert the analog signal to digital signal, as shown in Fig. 17. The attitude parameters monitored by each sensor are read by the controller.

### Single group of the advanced hydraulic support lifting process pose monitoring experiment

**Figure 18:**
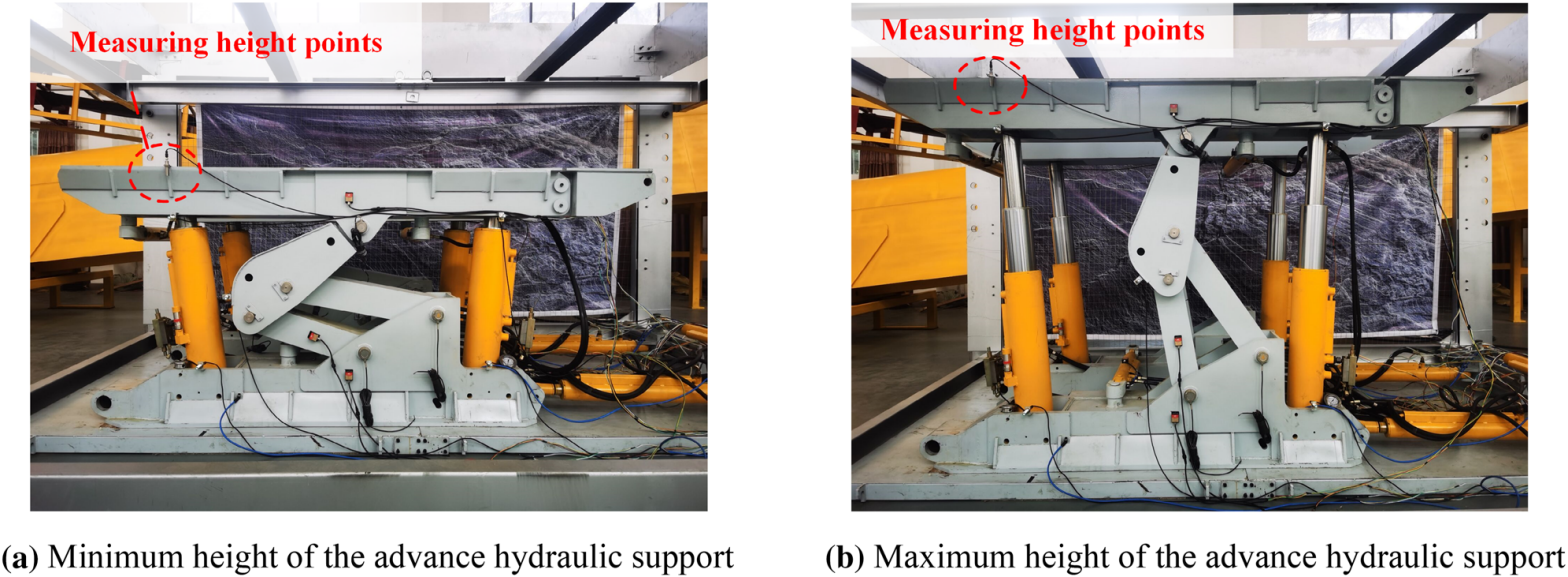
Advanced hydraulic support lifting process.

During the experiment, ultrasonic ranging sensors are installed at both ends of the bracket to monitor the distance between the measuring point of the top beam and the bottom plate of the experimental platform. The upper computer is manipulated for manual control. The bracket is increased from the lowest height to the highest height for 10 s and then decreased from the highest height to the lowest height for 10 s. The lifting process of the advanced hydraulic support is shown in Fig. 18.

The established attitude model is verified by comparing the front support height of the top beam and the back support height of the top beam of the single group of the advanced hydraulic support. The ultrasonic ranging sensor data and the acquisition frequency of the attitude sensor are unified to 10 Hz. In the experimental process, the data recorded in the experimental process are exported through the historical query module in the upper computer. Taking the front support height of the advanced hydraulic support as an example, the accuracy of the single group of support pose model proposed in this work is verified by comparing and analyzing 150 groups of monitoring data.

The support height data of the front end of the advanced hydraulic support are shown in Table 19, where *H_monitor* is the model to calculate the monitoring value, *H_true* is the real value collected by the sensor, and *H_error* is the error value.

**Table 19:** Front end support height data of the advanced hydraulic support.

Number	*H*_*true* (mm)	*H*_*monitor* (mm)	*H*_*error1* (mm)
1	1000	1004.66	4.66
2	1016.58	1021.24	4.66
3	1032.96	1037.59	4.63
4	1049.29	1053.89	4.6
5	1064.98	1069.56	4.58
$\cdots$	$\cdots$	$\cdots$	$\cdots$
71	1443.57	1443.52	−0.05
72	1440.95	1440.71	−0.24
73	1438.31	1437.92	−0.39
74	1435.75	1435.16	−0.59
75	1433.19	1432.41	−0.78
$\cdots$	$\cdots$	$\cdots$	$\cdots$
146	968.56	973.08	4.52
147	971.24	975.85	4.61
148	973.99	978.69	4.7
149	976.75	981.53	4.78
150	978.74	983.59	4.85

Based on the comparison of the support height, the maximum error between the calculated monitoring value and the measured real value is 4.85 mm, the minimum error is − 0.05 mm, and the average absolute error is 2.86 mm, which meet the accuracy requirements of the motion state monitoring of the advanced hydraulic support.

### Relative pose monitoring experiment of the advanced hydraulic support group

**Figure 19:**
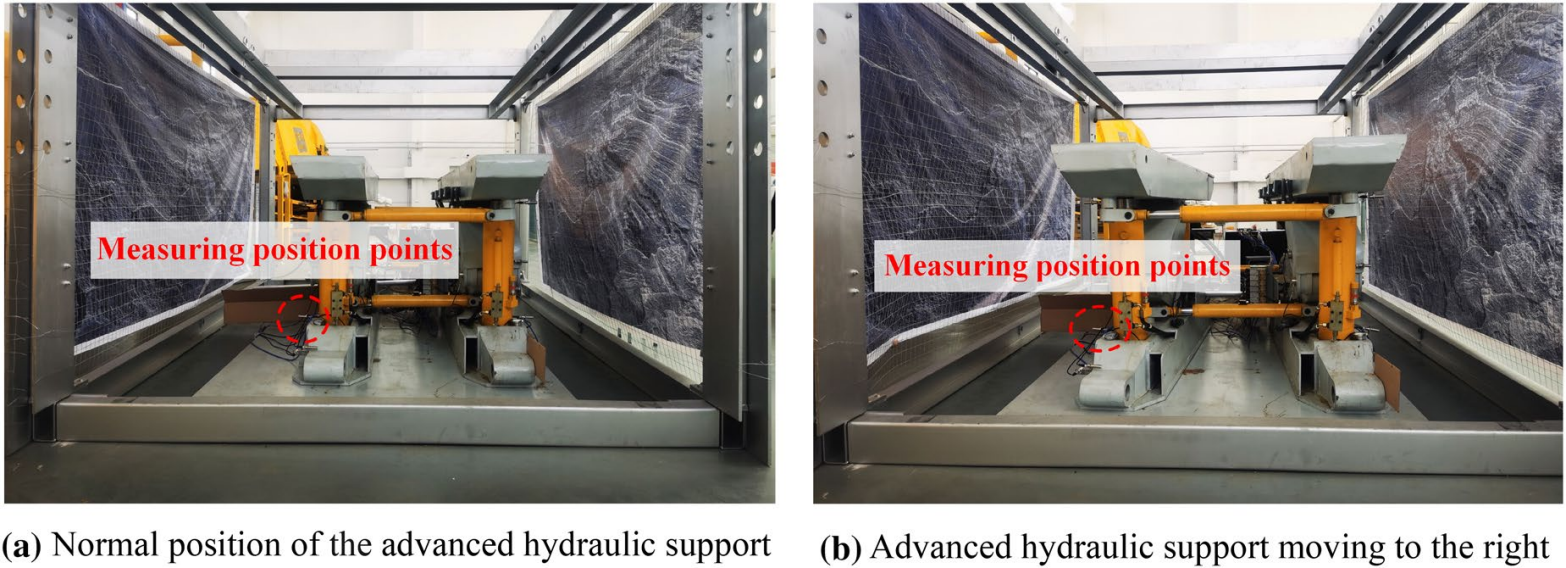
Advanced hydraulic support side push process.

The ultrasonic ranging sensor is installed at the front end of the support base to monitor the position of the measurement point and the reference coordinate system. The deformation of the surrounding rock of the simulated roadway is generated outward. The upper computer is manipulated to manually control the page and the balance cylinder to move to the right and adjust it to the normal support distance. The pushing process of advanced hydraulic support is shown in Fig. 19.

The monitoring method is verified by comparing the coordinates of the measuring point of the advanced hydraulic support, the expansion length, and the deflection angle of the base balance cylinder. During the experiment, the true values collected by the ultrasonic ranging sensor, the top beam attitude sensor, and the cylinder stroke sensor are extracted. The calculated monitoring value *C_monitor* and the measured real value *C_true* of the measuring point coordinates of the advanced hydraulic support are obtained through experiments, and the error *C_error* is calculated. The experimental data are shown in Table 20.

**Table 20:** Coordinate data of the measuring point of the advanced hydraulic support.

Number	*C*_*monitor*	*C*_*true* (mm)	*C*_*error* (mm)
1	(1485.54, 886.46)	(1481.31, 888.22)	(4.23, −1.76)
2	(1495.44, 885.41)	(1490.54, 887.17)	(4.90, −1.76)
3	(1503.96, 884.34)	(1499.76, 886.11)	(4.20, −1.77)
4	(1513.18, 883.24)	(1508.99, 885.01)	(4.19, −1.77)
5	(1522.41, 882.12)	(1518.23, 883.89)	(4.18, −1.77)
$\cdots$	$\cdots$	$\cdots$	$\cdots$
46	(1892.55, 821.31)	(1889.28, 823.07)	(3.27, −1.76)
47	(1901.82, 819.37)	(1898.59, 821.19)	(3.23, −1.82)
48	(1911.09, 817.47)	(1907.91, 819.29)	(3.18, −1.82)
49	(1920.36, 815.55)	(1917.21, 817.37)	(3.15, −1.82)
50	(1929.63, 813.61)	(1926.52, 815.44)	(3.11, −1.83)

In the above experiments, the maximum error between the calculated monitoring value of the coordinate $x$ axis of the measurement point and the measured real value is 4.90 mm, the minimum error is 3.08 mm, and the average absolute error is 3.71 mm. The maximum error between the calculated monitoring value of the coordinate $y$ axis of the measurement point and the measured real value is − 1.83 mm, the minimum error is − 1.76 mm, and the average absolute error is 1.78, which meet the accuracy requirements of the motion state monitoring of the advanced hydraulic support.

## Conclusions

1. The attitude state and deviation of the advanced hydraulic support in the working process are systematically analyzed. The D–H parameter matrix transformation method is used to establish the spatial attitude kinematic model of the single group of advanced hydraulic support and the advanced hydraulic support group. The pose parameters of each key node are obtained by global coordinate transformation.
2. The kinematic simulation analysis of the advanced hydraulic support group is carried out by ADAMS. The results of the comparison model show that the average absolute error of the support height calculation results of the single group of the advanced hydraulic support pose model is 3.92 mm, and the average absolute error of the coordinate calculation results of the moving monitoring point of the advanced hydraulic support group is (3.26 mm, 1.99 mm), verifying the accuracy of the model.
3. The accuracy of the established model is verified and analyzed by using the advanced hydraulic support attitude monitoring experiment platform. The maximum monitoring error is 4.9 mm, which further verifies the accuracy of the model and provides technical support for the adaptive support of the advanced coupling support system in coal mines.
4. The accuracy of the established model is verified and analyzed by using the advanced hydraulic support attitude monitoring experiment platform. The maximum monitoring error is 4.9 mm, which further verifies the accuracy of the model and provides technical support for the adaptive support of the advanced coupling support system in coal mines.

## Data Availability

Due to the ongoing project of the team, the data sets obtained during the current study are temporarily not public, but can be obtained from the corresponding authors according to reasonable requirements.
